# Locating and testing the healthy context paradox: examples from the INCLUSIVE trial

**DOI:** 10.1186/s12874-022-01537-5

**Published:** 2022-02-27

**Authors:** G. J. Melendez-Torres, Emily Warren, Obioha C Ukoumunne, Russell Viner, Chris Bonell

**Affiliations:** 1 Peninsula Technology Assessment Group, University of Exeter Medical School, Exeter, UK; 2 Department of Public Health, Environments and Society, London School of Hygiene and Tropical Medicine, London, UK; 3 NIHR Applied Research Collaboration South West Peninsula (PenARC), University of Exeter Medical School, Exeter, UK; 4 Department of Social Science, UCL Institute of Child Health, London, UK

**Keywords:** Cluster-randomised trials, Contextual effects, Mental wellbeing, Bullying, Intervention harms

## Abstract

**Background:**

The healthy context paradox, originally described with respect to school-level bullying interventions, refers to the generation of differences in mental wellbeing amongst those who continue to experience bullying even after interventions successfully reduce victimisation. Using data from the INCLUSIVE trial of restorative practice in schools, we relate this paradox to the need to theorise potential harms when developing interventions; formulate the healthy context paradox in a more general form defined by mediational relationships and cluster-level interventions; and propose two statistical models for testing the healthy context paradox informed by multilevel mediation methods, with relevance to structural and individual explanations for this paradox.

**Methods:**

We estimated two multilevel mediation models with bullying victimisation as the mediator and mental wellbeing as the outcome: one with a school-level interaction between intervention assignment and the mediator; and one with a random slope component for the student-level mediator-outcome relationship predicted by school-level assignment. We relate each of these models to contextual or individual-level explanations for the healthy context paradox.

**Results:**

Neither model suggested that the INCLUSIVE trial represented an example of the healthy context paradox. However, each model has different interpretations which relate to a multilevel understanding of the healthy context paradox.

**Conclusions:**

Greater exploration of intervention harms, especially when those accrue to population subgroups, is an essential step in better understanding how interventions work and for whom. Our proposed tests for the presence of a healthy context paradox provide the analytic tools to better understand how to support development and implementation of interventions that work for all groups in a population.

**Trial registration:**

Current Controlled Trials, ISRCTN10751359.

**Supplementary Information:**

The online version contains supplementary material available at 10.1186/s12874-022-01537-5.

## Background

Garandeau and Salmivalli [1] recently theorised the existence of a *healthy context paradox*. Using the example of school bullying interventions, they described that interventions that reduce the prevalence of victimisation (and thus improve overall rates of mental wellbeing) may actually worsen the mental wellbeing of those students who continue to experience victimisation during and after the intervention. Anti-bullying interventions may therefore strengthen rather than attenuate differences in mental wellbeing between victimised and non-victimised individuals. In this brief paper, we extend Garandeau and Salmivalli’s valuable contribution by: relating their work to our previous discussion of the need to consider dark logic models [2] theorising potential harms when developing interventions; formulating the healthy context paradox in a more general form defined by mediational relationships; reiterating that the healthy context paradox is a phenomenon that can only be detected in cluster-level interventions; proposing two statistical models for testing the healthy context paradox; and relating these statistical models to the meta-mechanisms (contextual and individual) that might be implicated in the healthy context paradox. We demonstrate these points using a mediational model from the INCLUSIVE trial [3], a school-randomised trial of a restorative practice intervention to prevent bullying and improve mental wellbeing amongst secondary school students in southeast England (see Table 1). Throughout this paper, our definition of mediation is classical; that is, a variable that explains part or all of the causal relationship between an independent variable and an outcome [4]. From an interventional perspective, a mediator is a variable on the causal path between an intervention and an outcome through which a significant indirect effect can be detected.

**Table 1:** The INCLUSIVE trial

We use data from INCLUSIVE [3], a school randomised trial of restorative practice in schools involving 40 schools (*n* = 6667 at baseline, *n* = 5960 at 36-month follow-up) serving students age 11-16 in south-east England from 2014 to 2017. Overall, the intervention, which comprised restorative practice, student participation in school decisions and a student social-emotional learning curriculum, was found to reduce student-reported bullying victimisation and improve mental wellbeing as well as benefit other secondary outcomes at 36-month follow-up for children aged 14-15 years. Full details of methods and overall results are presented elsewhere [3]. When we refer to the INCLUSIVE trial in terms of mediation, we use bullying victimisation as the mediator and mental wellbeing as the outcome.	

### The healthy context paradox and dark logic models

The healthy context paradox is a welcome contribution to the literature in that it provides intervention developers and implementers with additional insights into how school-based interventions might inadvertently cause harms. In our prior work on dark logic models, we described that harms could take the form of either *paradoxical effects*, wherein an intervention that seeks to improve an outcome in fact worsens it, or *harmful externalities*, wherein an intervention aiming to generate benefits in one domain generates harms in another [2]. As a heuristic, the healthy context paradox provides a way for intervention developers and evaluators to advance and refine intervention theory through understanding how interventions may not equally benefit all students [2, 5].

In relation to whole-school anti-bullying interventions, the healthy context paradox is an example of a paradoxical effect where the harm does not affect the entire study population (all students) but a subpopulation defined in terms of the intermediate effects (those who are bullied) of the intervention. That is, the harm affects a subpopulation defined in part by the impact of the intervention on the mediator. The healthy context paradox is also an *equity harm *[6], meaning that an intervention that improves health overall may worsen it for some, leading to exacerbations of existing inequalities between groups. For example, the INCLUSIVE theory of change suggested that schools where students know that their teachers are taking action to address bullying, and where being a bully goes against social norms, will have students with better mental wellbeing [7]. However, the healthy context paradox would suggest that students who are victimized may have worse mental health than before the intervention [1]. The healthy context paradox also suggests that this may be because they have fewer co-bullied peers to relate with and now suffer worse social isolation.

To generalise, implicit in the healthy context paradox is a specific mediational relationship defined by a psychosocial or behavioural mediator and a wellbeing outcome. In the study by Garandeau and Salmivalli [1], bullying victimisation is the mediator, but the mediator could be any similar variable capturing intermediate outcomes, for example bullying perpetration, which is also known to be linked to poor mental wellbeing; other forms of relational aggression, such as sexual harassment or dating and relationship violence; or even variables such as school commitment. The outcome of interest generally relates to mental wellbeing, but could hypothetically relate to any outcome where intervention effects on the outcome are mediated by intervention effects which make a behaviour or other experience less normative within a setting. The healthy context paradox thus relates to harms in wellbeing that affect a subpopulation that does not experience the benefits experienced by the broader study population.

In our original work on dark logic models [2], we proposed that a critical path through which intervention harms might arise is the interaction between the social structure within which an intervention is delivered and the agency of those interacting with the intervention, which may trigger unintended consequences. Such an interaction might also occur within the healthy context paradox. These processes might harm all those who continue to experience victimisation or those with particular vulnerabilities. Dark logic models for future school-based health interventions that seek to address bullying or other critical mediating behaviours should theorise potential adverse mechanisms that could operate at structural and individual levels and develop ways to measure the interaction between these.

### Understanding the healthy context paradox in relation to contextual effects

Implicitly but importantly, the healthy context paradox can be detected only in interventions that are allocated at the cluster level. That is, it is impossible to detect a healthy context paradox in a situation where an intervention is allocated at the individual level. It is important to stress that our focus is on *detection* of the healthy context paradox as opposed to its *generation*. The existence of an intervention-generated equity harm [6] of the type described above, specifically a general improvement in wellbeing arising from reductions in a behavioural or experiential mediator with a worsening in wellbeing for those who still report high levels of the mediator, might manifest but cannot be detected in an individually randomised trial. Consider, for example, if the INCLUSIVE trial had tested an intervention consisting only of individually-administered social-emotional learning without the school components, and was thus amenable of a trial that randomises individuals within schools. Even where this intervention is effective and the prevalence of bullying victimisation sharply declines, and victimisation becomes less normative, remaining victims may, for example, receive less support and experience worse mental health. While this equity harm exists in the same form as above, it cannot be identified as a *contextual* paradox because there is no basis to contrast cluster-level and individual-level impacts. This is despite the fact that in our hypothetical example, the equity harms generated by the intervention clearly worked through contextual, school-based mechanisms related to provision of support.

This conceptual basis for detecting the healthy context paradox in cluster randomised trials can be represented statistically, and these representations form the basis for proposing tests of the healthy context paradox. The rest of this section focuses on developing these representations using concepts from multilevel models, also known as generalised linear mixed-effects models or hierarchical linear models [8]. Multilevel models are frequently used in the analysis of cluster randomised trials as they can jointly consider the impact of cluster-level variables (such as treatment allocation) and individual-level variables (such as demographic characteristics) on outcomes [9]. In our example, clusters refer to schools in a trial, and individuals refer to students. First, we focus on how multilevel models estimate intervention impacts in cluster randomised trials; second, we consider how multilevel models can be used to identify contextual effects; and third, we reinforce why detection of the healthy context paradox can only occur in multilevel data structures, such as students nested within schools, specifically where interventions are allocated at cluster or school level.

### Estimating intervention impacts with multilevel models

When interventions are allocated at the cluster (or school) level, multilevel models use individual students’ reports of the study’s outcome to estimate differences between intervention and control schools through school-level means of those student reports [10]. Understood statistically, the healthy context paradox exists in the contradiction between school-level differences (between intervention and control groups) and student-level impact of an intervention (which may be more heterogeneous than a school mean can represent). Put otherwise, even if school-level means suggest that a school *on average* has experienced an improvement on wellbeing, it is possible that a minority of individual students within intervention schools experienced comparative worsening in their mental health, and that this worsening can be related to a specific individual characteristic or vulnerability. This heterogeneity in intervention effect forms the basis of the healthy context paradox.

### From cluster-level predictors to contextual predictors

This difference between school means and student impacts is an important first step in developing a statistical representation of the healthy context paradox. The next step is to understand how continuous predictors measured at the student level, such as mediators, can create both student effects and contextual school effects. In a multilevel modelling context, Raudenbush and Bryk [8] describe the contextual effect as the difference between the within-cluster coefficient (relationship of an individual value of a predictor and an individual value of an outcome within a cluster) and the between-cluster coefficient (relationship between cluster-level mean of a predictor and cluster-level mean of an outcome) for a predictor with an outcome. While contextual effects exist anywhere individuals are grouped in clusters (i.e. students grouped in schools), we can also describe contextual effects as follows: is there an impact of a school-mean predictor on an outcome above and beyond the student-level relationship between predictor and outcome? Their classic example [8, 11] relates socioeconomic position to performance on standardised tests. Socioeconomic position can be measured at the student level, with individual students’ reports, and also at the school level, with the average of students’ reports. To restate this example as a question: is there an impact on students’ test scores, above and beyond the student-level associations between socioeconomic position and test scores, of studying in a school with low average socioeconomic position?

Contextual effects can only be detected where predictors are measured at the individual level and can be aggregated to cluster-level means, leading to simultaneous testing of both the association between the variable measured at the individual level and the outcome and the association between the variable aggregated to the cluster level and the outcome. As a result, contextual effects are not relevant for intervention allocation status as that is only a cluster-level variable, but rather contextual effects are relevant for understanding the association between mediators and outcomes.

### Detection of the healthy context paradox in multilevel data structures

 To be clear, this is not to say that the healthy context paradox reduces to testing a contextual effect, as we discuss below. Instead, a necessary precondition to understanding the healthy context paradox is to parse individual-level and cluster-level variation in the relationship between a predictor and an outcome and therefore identify a contextual effect. This is because, consistent with the range of mechanisms identified by Garandeau and Salmivalli [1], the intervention could potentially influence (a) the school context within which the link between bullying and mental wellbeing occurs or (b) students’ experiences of how bullying links to mental wellbeing even where school-level contexts have improved. For example, INCLUSIVE may have changed school culture or it may have worsened a student’s bullying even when bullying has become less prevalent. Thus, not only conceptually but statistically, because contextual effects can only be directly measured in multilevel data structures, the healthy context paradox can only be detected in cluster-allocated, or school-allocated, interventions.

## Methods

### Testing for the healthy context paradox

**Fig. 1:**
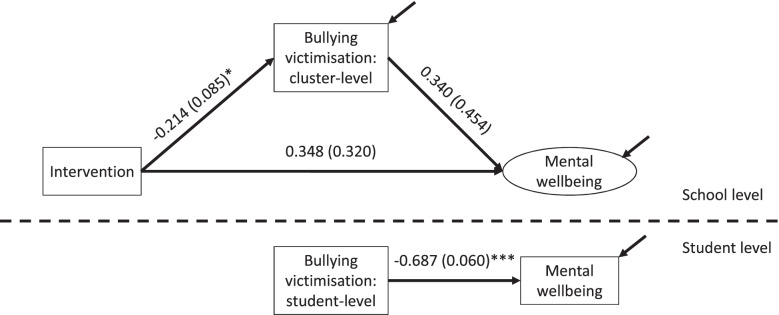
Baseline mediation model

Putting this all together, any test of the healthy context paradox requires the decomposition of the intervention’s mediational pathways into mediational pathways at both school and student levels. The insight of Raudenbush and Bryk [8] about the estimation of contextual effects has been influential in understanding mediation in cluster randomised trials. The healthy context paradox corresponds to a specific type of mediation known as cross-level mediation, and specifically 2-1-1 mediation [12]. The 2-1-1 mediation model exists when an intervention is allocated at level 2, or at the school level; influences a level 1, or student, mediator (e.g. bullying); and also influences a level 1 outcome (e.g. mental wellbeing). Of note is that both the mediator and the outcome can be measured at the student level and aggregated at the school level to generate school-level means. Pituch and Stapleton [11] have observed that specific approaches to testing cross-level mediation generate greater power and greater insights in distinguishing between the impact of the intervention on the individual and cluster levels; that is, a school-randomised intervention may effect a specific mediational pathway both through the student-level relationship between mediator and outcome and through a school-level contextual effect which modifies the nature of this student-level relationship. To parse these relationships, they suggest testing a mediational model with the school-level paths between intervention and school-level mediator mean, intervention and school-level outcome mean, school-level mediator mean and school-level outcome mean; and with the student-level path between the mediator and the outcome (see Fig. 1). In this formulation, the school-level mediator-outcome relationship is therefore the contextual effect (or the effect of the school level of the mediator), the student-level mediator-outcome relationship is the individual effect (or how a student’s report of the mediator links to a student’s report of the outcome), and the sum of the school-level and student-level mediator-outcome coefficients multiplied by the coefficient relating mediator and intervention is the total indirect effect. Importantly, in the first instance, this requires including an uncentred (i.e. at its original value) mediator at the student level alongside a school-level mean for the mediator.

Drawing on this 2-1-1 mediation model, we propose that what Garandeau and Salmivalli [1] describe as a moderated mediation model is better described as a special case of mediation where the intervention through its school-level effects moderates the relationship between mediator and outcome. This is because moderated mediation is most generally understood as a situation where a fourth variable explains heterogeneity across the trial sample in the magnitude of the indirect effect [13]. However, if the healthy context paradox is an equity harm generated by a cluster-level intervention, then the intervention itself cannot be that fourth variable and cannot moderate the link between intervention and mediator. The intervention can, however, moderate the link between mediator and outcome. This will be familiar to those approaching mediation from the potential outcomes framework as a treatment-by-mediator interaction [14].

If the general form of the healthy context paradox is treatment-by-mediator interaction and the mediator-outcome relationship can be measured at both individual and cluster levels in cluster-randomised trials, it follows that there are two potential treatment-by-mediator interactions to be estimated: one at the cluster level, in which the contextual effects are moderated by intervention; and one at the individual level, in which individual effects are moderated by intervention. We propose tests of each of these below as Test 1 and Test 2 respectively, and provide indicative code for implementation in Mplus (see Appendix 1). Both of these treatment-by-mediator interactions provide a test for the existence of the healthy context paradox, and are suggestive of different possible meta-mechanisms for the paradox’s existence in a given trial.

The mediation models we develop draw on two key study outcomes: bullying victimisation assessed using the Gatehouse Bullying Scale [15], at 36-month follow-up, and a measure of functional and psychological mental wellbeing, the Short Warwick-Edinburgh Mental Wellbeing Scale or SWEMWBS [16], at 36-month follow-up. Higher scores on the Gatehouse Bullying Scale represent higher levels of victimisation, while higher scores on the SWEMWBS represent higher levels of mental wellbeing. We restrict our consideration here to mediators and outcomes as measured on linear scales; estimation of direct and indirect effects is more complicated where either mediator or outcome requires a different, non-normal link function [17].

### A baseline mediational model from INCLUSIVE

To estimate this model, we use the 2-1-1 model described above, including regressing the school-level mediator mean on intervention status, the school-level outcome mean on the school-level mediator mean and intervention status, and the student-level outcome on the student-level mediator. This accomplishes the separation of contextual and individual effects in the mediator-outcome relationship.

We note at this point that a non-significant relationship between mediator and outcome at either school or student level should not preclude undertaking either Test 1 or Test 2, as, for example, the average of two effects could produce a misleading null effect overall. However, a non-significant path between intervention and mediator suggests that the candidate mediator should not be considered further.

### Test 1: contextual effects

To estimate this model, the relationship between mediator and outcome at the school level is moderated by intervention status. This is an extension to the standard structural equation model-based mediation method, where the interaction between intervention allocation and the mediator score is entered as an additional predictor of the outcome [14]. Thus, the findings from this model will generate different estimates of the contextual effect between mediator and outcome. A standard significance test can be used on the interaction term to test for differential contextual effects on the intervention arising from a moderated relationship between mediator and outcome.

Where a healthy context paradox is present at a contextual level, the results of the test will indicate that a contextual effect for the mediator-outcome relationship has a magnitude indicating less benefit (or greater harm) in the intervention as compared to the control group, even where the intervention-mediator relationship suggests a meaningful and positive impact of the intervention. The interpretation of this is that the intervention may have triggered structural mechanisms that are linked to a worsening of school context for those who experience bullying; but also for those who do not. This is because, in this circumstance, the intervention reduces levels of bullying at the school level; may still improve mental wellbeing overall at school level, including through direct effect on the outcome; but potentiates a worsening school-level link between bullying and mental health, so that intervention schools with higher levels of bullying experience an even greater negative contextual impact on average levels of mental wellbeing. This may be enough to outweigh positive benefits from the intervention at individual and contextual levels, because intervention schools with higher levels of bullying have an even larger association with worsening school mental health than control schools.

### Test 2: individual effects

To estimate this model, the relationship between mediator and outcome at student level is moderated by intervention status. This is a standard random slope model where the student-level relationship is moderated by a school-level variable, here intervention status [11]. A direct test of significance is usually available for this relationship. However, a complication of this model is that to estimate this relationship without bias, the individual-level mediator must be centred within schools [18]. This means that the school-level relationship between mediator and outcome is no longer the contextual effect alone but rather the sum of the contextual and individual effects [11]. While this is not a barrier to testing, it should be borne in mind in interpretation, as in this model the value of the school-level relationship between mediator and outcome may be closer to the sum of the student-level and school-level paths in the baseline mediation model. As with most random slope models, it can be useful to co-vary the random slope component with the intercept for the dependent variable.

Analogous to above, where a healthy context paradox is present at the student level, the results of the test will indicate that the student-level relationship between mediator and outcome has a magnitude indicating less benefit (or greater harm) in the intervention as compared to the control group, even where the intervention-mediator relationship is significant. The interpretation of this is that even as the intervention improves scores on the mediator and on the outcome overall for students, those students experiencing worse values for the mediator (e.g. bullying) also experience proportionally worse and more inequitable values for the outcome (e.g. mental wellbeing).

## Results

### Baseline mediation model

Our baseline model (see Fig. 1) showed that the impact of the intervention on mental wellbeing was mediated by improvements in bullying victimisation. However, these improvements were mediated at the student level (β =-0.687, SE=0.060) without a significant school-level contextual effect. That is, the school-level path from victimisation to mental wellbeing (β = 0.340, SE=0.454) was not significant. Because a mediation pathway was also included at the student level, the school-level path represents the contextual effect of the mediator. The interpretation of this model is that part of INCLUSIVE’s beneficial effect on mental wellbeing was through reducing victimisation; but that the link between victimisation and mental wellbeing can be understood in this baseline model at the student level (students with lower victimisation on average had better mental wellbeing) without a contextual effect at the school level (beyond the student-level relationship, schools with lower victimisation did not have students with better mental wellbeing on average).

### Test 1 in INCLUSIVE

We examined the interaction between the school-level mean of the mediator, bullying victimisation, and intervention allocation status and entered this as a third predictor of the outcome. The function of this predictor, as discussed above, is to induce a different value of the contextual effect of the mediator on the outcome depending on intervention status (i.e., a different value for each of the intervention and control arms).

**Fig. 2:**
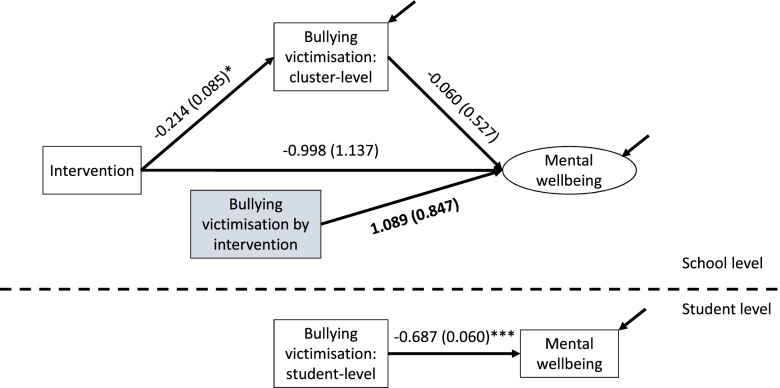
Testing for contextual effects

As in the baseline model, there is a significant and meaningful mediational pathway from intervention to outcome (see Fig. 2). However, the test for contextual effects in the healthy context paradox suggests that this paradox is not supported in INCLUSIVE, and a Wald test did not find that this model was significantly different from the baseline model (*df*=1, *p*=0.20). Neither the interaction of intervention with mediator (β = 1.089, SE=0.847) nor the direct path at the between level from mediator to outcome (β=-0.060, SE=0.527) were significant. Indeed, the intervention by mediator interaction, while non-significant, would be interpreted as having an opposite effect; namely an important enhanced effect on mental wellbeing of the intervention in schools experiencing residually higher rates of bullying victimisation. In short, applying test 1 to data from INCLUSIVE does not suggest that the intervention worsened the link at school level from bullying victimisation to decreased mental wellbeing. Were this to have been the case, we would have expected the intervention by mediator interaction to have a significant effect with a negative sign.

### Test 2 in INCLUSIVE

In addition to the ‘baseline’ mediation model, we: (a) cluster mean-centred the mediator (that is, redefined student-level scores on bullying victimisation as deviations from the school-level mean), (b) specified a random slope component for the relationship between student-level mediator and the outcome, and (c) regressed this random slope component on intervention status at the school level. The function of point c) is to determine a different value of the individual-level relationship between the mediator and the outcome depending on intervention status.

**Fig. 3:**
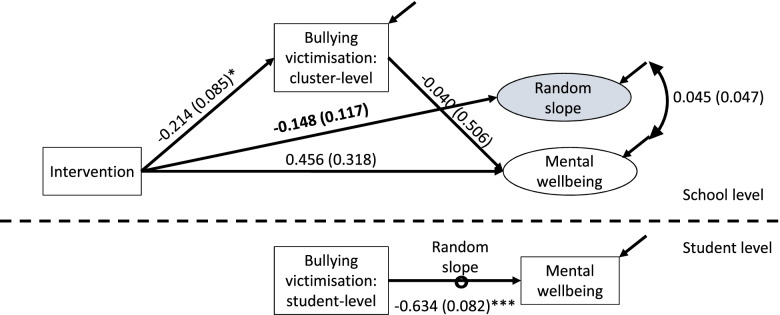
Testing for individual effects

Again, a significant and meaningful mediational pathway from intervention status to mental wellbeing persists (Fig. 3). However, in this analysis, the student-level relationship between bullying victimisation and mental wellbeing is regressed on intervention status. Thus, the baseline estimate of the relationship at the student level between the mediator and the outcome (β=-0.634, SE=0.082) properly refers to the mediator-outcome relationship in students in control schools. The regression of student-level slope on intervention thus yields the difference between intervention and control groups on the relationship between student-level mediator and outcome *(*β=-0.148, SE=0.117). The interpretation of this coefficient is that in intervention schools, the relationship between bullying victimisation and mental wellbeing is stronger; that is, students experiencing victimisation experience an even greater decrement in mental wellbeing, consistent with the healthy context paradox. However, this path is not significant and thus the model does not support the existence of the healthy context paradox at individual level. A Wald test did suggest that this model was significantly different from a baseline model (*df*=3, *p*=0.03); however, this was due to a significant random slope component for the student-level mediator-outcome relationship. A Wald test comparing this model to a model with no relationship between student-level slope and intervention status and with no covariance between slope and random intercept did not support the existence of the healthy context paradox (*df*=2 *p*=0.29).

## Discussion

Of the two proposed tests, Test 2 is probably closest to how Garandeau and Salmivalli understood the healthy context paradox. However, we believe that Test 1 is important as well. This is because it sheds light on potential school-level structural explanations for the healthy context paradox—structural explanations that form an important part of the theoretical basis for this paradox—and can account for exacerbation in differences *between intervention and control schools* in the mental health of those experiencing victimisation. In contrast, Test 2 sheds light on exacerbation in differences *within intervention schools* in the mental health of those experiencing victimisation. To the extent that understanding mechanisms in interventions is an inductive task, the results of each test provide analytic purchase in inferring the mechanisms for evidenced health inequalities both between schools and within school, suggesting that these are either primarily contextual or primarily individual, or both. These two tests thus relate to two different meta-mechanisms, structural and individual, that can drive the existence of the healthy context paradox. Given the increasing focus on complex systems approaches to evaluation [19], understanding how interventions work over multiple systems of influence can help in developing intervention theory. The healthy context paradox may also be useful in other areas of public health that seek to reduce the frequency or prevalence of specific population characteristics or behaviours, thus stigmatising those who are ‘left behind’ by the intervention. For example, the healthy context paradox could be tested for interventions targeting diet and physical activity, where interventions that stigmatise overweight can worsen contextual or individual relationships between overweight and mental wellbeing; or interventions that seek to reduce sexual risk, thus stigmatising those who continue to engage in risk behaviours.

## Conclusions

We are grateful to Garandeau and Salmivalli for this important contribution to the understanding of how school-level interventions may not equally benefit all students. Greater exploration of intervention harms, especially when those accrue to population subgroups, is an essential step in better understanding how interventions work and for whom, and thus in supporting the decision to implement interventions in contexts different from the ones where interventions may have been originally evaluated.[20] Our proposed tests for the presence of a healthy context paradox provide the analytic tools to better understand how to make school contexts effective places for all children and young people to reach their full potential.

## Supplementary Information

**Additional file 1.** Model code for implementation in Mplus

## Data Availability

The dataset analysed in this study relates to the INCLUSIVE trial. It is not publicly available as it contains sensitive information about trial participants who were below the age of majority at the time of the intervention.

## Abbreviations

SE: Standard error.
SWEMWBS: Short Warwick-Edinburgh Mental Wellbeing Scale.
