# Supplementary material for: Locating and testing the healthy context paradox: examples from the INCLUSIVE trial
Source: BMC Med Res Methodol. 2022 Feb 27;22:57. doi: 10.1186/s12874-022-01537-5 (PMC8883633; doi:10.1186/s12874-022-01537-5)
Supplement: Supplementary file 1 — Additional file 1. Model code for implementation in Mplus [file 12874_2022_1537_MOESM1_ESM.docx]

**Appendix 1.** Model code for implementation in Mplus

**Baseline model**

Variable gbs3m is the school-level mean of the mediator at the 24-month follow-up, while variable gbs3 is the student-level value of the mediator. Variable mwb3 is the individual-level value of the outcome at the 36-month follow-up, with a random intercept at between-level.

Data:

File is "trial.dat";

Variable:

Names are

school gbs3 mwb3 intervention gbs3m;

MISSING are all (-999);

USEVARIABLE are gbs3 mwb3 intervention gbs3m;

WITHIN is gbs3;

BETWEEN is intervention gbs3m;

CLUSTER is school;

Analysis:

TYPE is TWOLEVEL;

Model:

%WITHIN%

mwb3 ON gbs3;

%BETWEEN%

gbs3m mwb3 ON intervention;

mwb3 ON gbs3m;

**Test 1: contextual effects**

The newly defined variable xm is the intervention-by-mediator interaction.

Data:

File is "trial.dat";

Variable:

Names are

school gbs3 mwb3 intervention gbs3m;

MISSING are all (-999);

USEVARIABLE are gbs3 mwb3 intervention gbs3m xm;

WITHIN is gbs3;

BETWEEN is intervention gbs3m xm;

CLUSTER is school;

Define:

xm = intervention * gbs3m;

Analysis:

TYPE is TWOLEVEL;

Model:

%WITHIN%

mwb3 ON gbs3;

%BETWEEN%

mwb3 ON intervention;

gbs3m ON intervention;

mwb3 ON gbs3m;

mwb3 ON xm;

**Test 2: individual effects**

Variable sb is the random slope component for the relationship between the individual mediator and the individual outcome. In addition, for this analysis, the mediator gbs3 has been centred at the group mean for all schools.

Data:

File is "trial.dat";

Variable:

Names are

school gbs3 mwb3 intervention gbs3m;

MISSING are all (-999);

USEVARIABLE are gbs3 mwb3 intervention gbs3m;

WITHIN is gbs3;

BETWEEN is intervention gbs3m;

CLUSTER is school;

Define:

CENTER gbs3 (GROUPMEAN);

Analysis:

TYPE is TWOLEVEL RANDOM;

Model:

%WITHIN%

sb | mwb3 ON gbs3;

%BETWEEN%

gbs3m mwb3 ON intervention;

mwb3 ON gbs3m;

sb ON intervention;
